# Search of Allosteric Inhibitors and Associated Proteins of an AKT-*like* Kinase from *Trypanosoma cruzi*

**DOI:** 10.3390/ijms19123951

**Published:** 2018-12-08

**Authors:** Rodrigo Ochoa, Cristian Rocha-Roa, Marcel Marín-Villa, Sara M. Robledo, Rubén E. Varela-M

**Affiliations:** 1 PECET, Facultad de Medicina, Universidad de Antioquia, Medellín 050010, Colombia; rodrigo.ochoa@udea.edu.co (R.O.); marcel.marin@udea.edu.co (M.M.-V.); sara.robledo@udea.edu.co (S.M.R.); 2 GEPAMOL, Centro de Investigaciones Biomédicas, Universidad del Quindío, Armenia 630004, Colombia; ccrochar@uqvirtual.edu.co; 3 QUIBIO, Facultad de Ciencias Básicas, Universidad Santiago de Cali, Cali 760035, Colombia

**Keywords:** *Trypanosoma cruzi*, protein kinase B, pleckstrin homology domain, drug discovery

## Abstract

Proteins associated to the PI3K/AKT/mTOR signaling pathway are widely used targets for cancer treatment, and in recent years they have also been evaluated as putative targets in trypanosomatids parasites, such as *Trypanosoma cruzi*. Here, we performed a virtual screening approach to find candidates that can bind regions on or near the Pleckstrin homology domain of an AKT-*like* protein in *T. cruzi.* The compounds were also evaluated in vitro. The in silico and experimental results allowed us to identify a set of compounds that can potentially alter the intracellular signaling pathway through the AKT-*like* kinase of the parasite; among them, a derivative of the pyrazolopyridine nucleus with an IC_50_ of 14.25 ± 1.00 μM against amastigotes of *T. cruzi*. In addition, we built a protein–protein interaction network of *T. cruzi* to understand the role of the AKT-*like* protein in the parasite, and look for additional proteins that can be postulated as possible novel molecular targets for the rational design of compounds against *T. cruzi*.

## 1. Introduction

Annually, around 40,000 new cases of people with Chagas disease are reported. This pathology is caused by a flagellated protozoan called *Trypanosoma cruzi*, which is transmitted to humans through a triatomine insect that functions as its vector. This parasite has two obligatory phases in the host, a replicative intracellular form called *amastigote* and a non-reproductive blood form called *trypomastigote*. Some of the effects of Chagas disease are alterations in the immune response, cardiomyopathy, and gastrointestinal disease. Two antiparasitic drugs, nifurtimox and benznidazole are used for treatment, but these have shown deficiencies associated to their toxicity and low effectiveness in the chronic phase, as well as reported cases of drug resistance [1,2,3]. Therefore, it is necessary to search for new, effective, and safe pharmacological alternatives against Chagas disease.

One strategy to design novel inhibitors is through the identification of novel molecular targets. For example, a subgroup of Ser/Thr kinases proteins called AGC kinases has been described as crucial for the regulation, growth, survival, differentiation, metabolism, and proliferation of cells. The protein kinase B (PKB or AKT) is part of this group [4,5]. There are numerous studies of the inhibition of PKB proteins and those present in the PI3K/AKT/mTOR signaling pathway, in order to control malignant cells and find better therapeutic alternatives for different types of cancer [6,7,8,9]. Some proteins from the PI3K/AKT/mTOR pathway are also studied for the control of some parasitic pathology. Recently, we reported our studies of recognition and inhibition of the *Ld*-RAC/AKT-*like* protein in *Leishmania panamensis*, suggesting that the AKT-*like* protein in *Leishmania* spp. could be a promising therapeutic target for treatments against Leishmaniasis [10,11,12,13].

The first report of PKB proteins in *T. cruzi* was made by Pascuccelli et al. in 1999. This protein is released in the three stages of the parasite life cycle, *epimastigote* (form present in the insect vector), *amastigote*, and *trypomastigote*, making it a promising drug target for the treatment of *T. cruzi* [5,14]. This protein contains in its amino terminus region a highly conserved domain called a Pleckstrin homology domain (PH), which briefly regulates the transport of crucial substrates (PI2P and PI3P) for the effective downstream signaling of proteins present in the PI3K/AKT/mTOR pathway. Therefore, this domain can be used to potentially control the PKB protein activity [15,16].

In recent years, computational methodologies have been used for drug discovery purposes in different diseases. Approaches such as virtual screening and molecular simulations, among others, allow the prediction of possible interactions between ligands and a receptor [17,18]. Here, we implemented computational tools to identify new compounds with possible preferences for pockets near the PH domain present in AKT-*like* proteins of *T. cruzi*. In addition, we built a potential protein–protein interaction network to understand the possible effects of inhibiting this protein in the parasite, and the identification of additional potential targets. Finally, we validated through in vitro assays some of the novel compounds with promising anti-Chagas activity.

## 2. Results

### 2.1. Modeling and Prediction of Pockets in the AKT-Like Protein of T. cruzi

**Figure 1:**
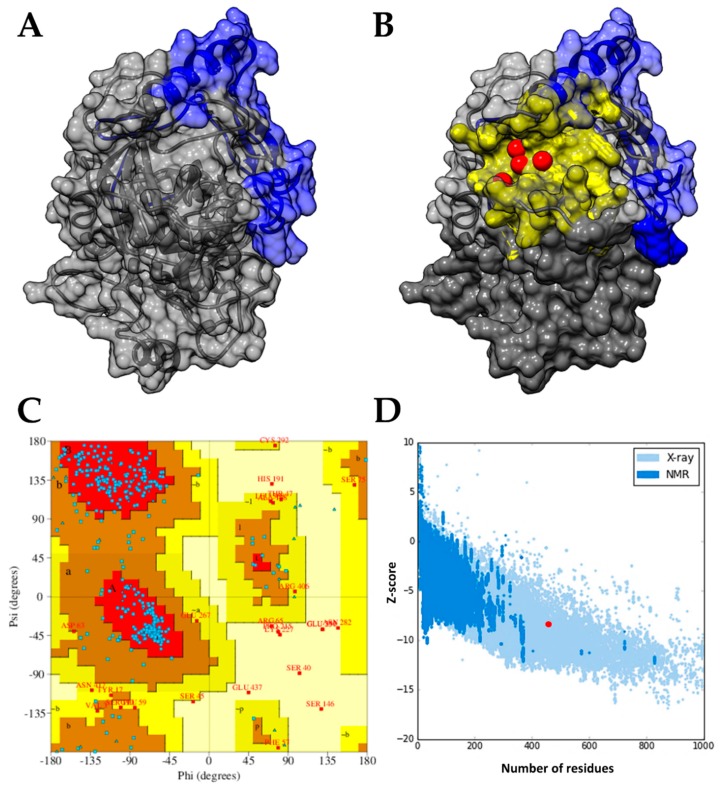
(**A**) Structural model chosen of the *Tc*AKT-*like* protein; the Pleckstrin homology (PH) domain is shown in blue, while the rest of the protein is shown in gray in a schematic way. (**B**) Selected drug pockets located near the PH domain of *Tc*AKT-*like* predicted by the PockDrug (yellow surface) and metaPocket (red spheres) tools. (**C**) Analysis of the stereochemistry (angles Ψ and Φ) of the model selected for the *Tc*AKT-*like* protein; 95% of the residues are in favored and allowed regions. (**D**) Evaluation of the overall quality of the *Tc*AKT-*like* model; the model presented a Z-score (−8.42, highlighted with a red dot) similar to the structures of the same size resolved experimentally.

The model obtained for the *Tc*AKT-*like* protein showed conserved structural folding between protein kinases. It contains the PH domain, which has a length of 99 amino acids, from Ser2 to Pro100 (Figure 1A). The stereochemistry of the model was examined with the Ramachandran plot, which suggests that 95% of the residues are in the favorable and allowed regions (Figure 1C). The overall quality of the model was evaluated with the Z-score. Figure 1D shows the Z-score values for all proteins determined experimentally with X-ray and NMR spectroscopy; the value obtained for the *Tc*AKT-*like* protein model was −8.42, which is within the range of typical values for structures experimentally determined of the same amino acid length. These results suggest adequate folding and acceptable structural quality to continue with the drug discovery pipeline.

To detect possible pockets located near or on the PH domain of the *Tc*AKT-*like* protein, the PockDrug and metaPocket web servers were used. The metaPocket results are the consensus of eight prediction tools, and in our case suggest a high probability that the pocket regions detected are potential ligand-binding sites. Four of the five predicted pockets were found located in the same superficial slit of the protein, near to the PH domain. These pockets are shown as red spheres in Figure 1B and represent the center of each predicted pocket [19]. The PockDrug web server allows the characterization of the predicted pockets and introduces a Druggability score. For the selected pocket, we obtained a Druggability score of 0.86 (from 0 to 1), suggesting a high probability of being a ligand-binding site in the *Tc*AKT-*like* protein. The pocket predicted by PockDrug that is closer to the PH domain is shown in Figure 1B. This pocket is composed of approximately 57 residues, with a volume, diameter, and radius of 6620 Å^3^, 36.4 Å, and 18.2 Å, respectively.

### 2.2. Virtual Screening and Ligand Property Predictions

**Figure 2:**
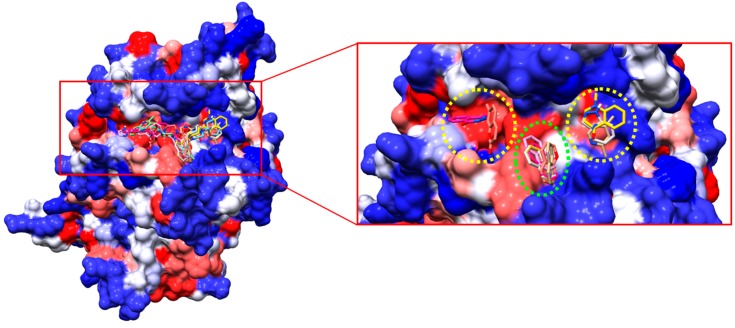
Selected compounds of the virtual screening coupled in the predicted binding site for the *Tc*AKT-*like* protein (**left**). Similar structural fragments between the coupled compounds. The naphthalene fragment could function as an anchor for each compound (**right**).

After the virtual screening, we obtained the best 1000 compounds, and the first 8 were selected for in silico structure activity relationship analysis and validation using in vitro assays. In addition, the compounds were subjected to a second molecular docking program as stated in the Methods section. The results suggest a similar trend in the affinity of the compounds for the predicted binding site, sharing also a similar ranking (Table 1). Figure 2 shows all the compounds docked to the *Tc*AKT-*like* protein in the pocket region near the PH domain.

Visual inspection allowed us to find shared fragments among all the docked compounds. For example, compounds with heterocycles and aromatic rings with heteroatoms were mostly docked in the region demarcated in yellow circles (Figure 2). Groups such as naphthalene were docked in the region demarcated in green. This information can be taken as a starting point for further studies on the rational design of drugs based on the structure of the receptor.

Regarding the prediction of toxicological risk, an extensive report is available in the Supplementary information (Table S1), and a summary report is available in Table 1. The toxicity prediction results suggest that compounds **UBMC-5**, **UBMC-6**, and **UBMC-8** would not present toxicological risks in future in vitro or in vivo experiments, while the other compounds are potentially associated with toxicological risks.

### 2.3. Results of the In Vitro Assay

The eight compounds evaluated had different effective IC_50_ concentrations, highlighting the compound **UBMC-6** with 14.25 ± 1 μM, followed by the compound **UBMC-8** with 18.26 ± 1.30 μM and **UBMC-7** with 19.44 ± 0.35 μM; these three compounds had an effective concentration with high activity. However, their cytotoxicity (LC_50_) was considered high. The compounds **UBMC-1** and **UBMC-3** presented an IC_50_ of 34.92 ± 3.87 and 37.53 ± 2.65 μM, respectively, with moderate activity and high cytotoxicity, except for the compound **UBMC-3** that was considered potentially noncytotoxic. Finally, compounds **UBMC-2**, **UBMC-4**, and **UBMC-5** presented IC_50_ concentrations of 62.44 ± 4.62, 72.13 ± 8.58, and 69.82 ± 9.23 μM, respectively, which are the ones with the lowest activity and high cytotoxicity.

Although the order of the compounds according to the in vitro results (Table 2, from lowest to highest IC_50_ value) does not match the order of molecular docking results (Table 1, from lowest to highest binding score predicted in kcal/mol), all the compounds evaluated had in vitro activities below 75 μM. It should also be mentioned that the differences between the scores predicted by molecular docking are not large enough to correlate them to their in vitro activities, as evidenced with compound **UBMC-6** that reported better in vitro activities than compounds such as **UBMC-3**, **UBMC-4**, and **UBMC-5**.

### 2.4. Role of the AKT-Like Protein in a Reconstructed Protein–Protein Interaction Network of T. cruzi

**Figure 3:**
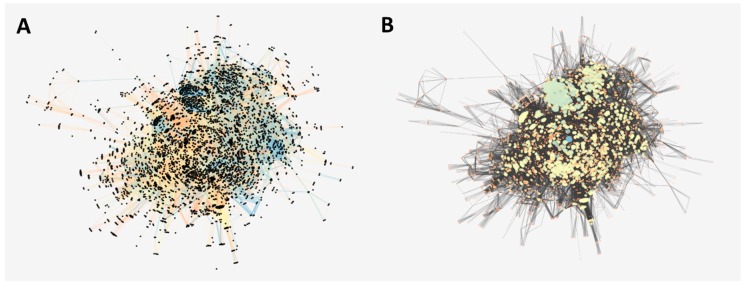
(**A**) Interaction network of *T. cruzi* strain CL Breiner. The nodes (proteins) are presented in black and the score associated with the interaction is presented in colors, blue being more reliable than orange. (**B**) Interaction network of *T. cruzi* strain CL Breiner. The axes (interactions) are represented in black and on a scale of orange (less connected) to blue (more connected) the nodes (proteins) of the network. The size of the circle indicates its degree of connectivity (degree).

In order to understand, from a biological perspective, the role of the *Tc*AKT-*like* protein, we reconstructed as much as possible the complete interactome of *T. cruzi*. We obtained a total 19,242 *T. cruzi* proteins derived from its whole-genome shotgun (WGS) genome project [20]. Then, we looked for all the inferred and validated interactions. Those with scores higher than 700 were filtered (Figure 3A). After that, the network represented 4910 proteins with 132,905 interactions.

The network reports a scale-free topology, indicating that highly connected proteins are located in the central region, as indicated in Figure 3B. After analyzing the degree of connectivity by number of nodes, we found that this distribution had a linear tendency behavior with a negative slope, with a R^2^ of 0.735 (unpublished figure). Although this is not a high value compared to other networks (probably due to lack of coverage), it is a sufficient condition to proceed with posterior topological calculations. We also observed a large number of nodes with a high degree of connectivity (Figure 3B).

### 2.5. Identification of Proteins Potentially Involved in the AKT-Like Pathway from the Parasite

**Figure 4:**
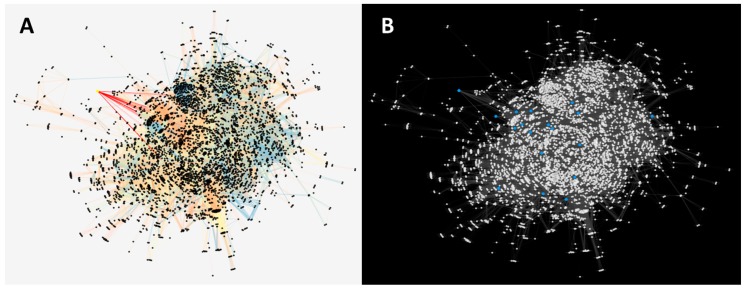
(**A**) Protein–protein interaction network with the AKT-*like* protein signaled in yellow, where the axes that connect with the closest neighboring nodes (located in the center of the network) are highlighted in red. (**B**) Protein–protein interaction network with proteins represented as white nodes, and in blue are those that were mapped with the PI3K/AKT/mTOR pathway of human proteins.

After obtaining a list of human proteins associated to the PI3K/AKT/mTOR pathway, we mapped them with proteins included in the protein–protein interaction network of *T. cruzi*, including *Tc*AKT-*like* (Figure 4A). The complete results are reported in Supplementary Table S3. Topological properties were calculated, where the *Tc*AKT-*like* yielded a degree value of 89 and a betweenness centrality of 0.002, which can be associated to a pleiotropic nature. The other proteins were also analyzed and visualized as blue nodes in Figure 4B. The proteins with their respective calculations of degrees of connectivity (degree) and betweenness centrality are reported in Table S4.

Most of the proteins reported connectivity data within the mean and are also relatively central to certain pathways within the network. Particularly, we found two orthologs of the AKT3 and AKT2 human proteins, where one of them (highlighted in bold in Table S4) presents the conserved regions of the predicted AKT-like (including the PH domain), with an additional zinc finger domain, which can be also potentially explored as another allosteric site of the protein in T. cruzi. The topological calculation, after excluding the proteins associated with ribosomal units let us obtain a ranking of 30 proteins with the highest values. This list (Supplementary Table S5) provides interesting information for finding new pharmacological targets against T. cruzi. Among them, we found DNA topoisomerase 2 proteins [21], GMP synthase [22] and dihydrofolate reductase–thymidylate synthase [23], which have been previously studied as molecular targets for the treatment of Chagas disease.

## 3. Discussion

In recent years, there has been interest in postulating drug targets in parasites that has been related to gene expression, cytoskeleton, membrane integrity, intermediates in metabolism, and intracellular signaling, which is the case of the AKT-*like* protein in *T. cruzi*. There is information that shows the relationship of proteins such as PI3K and AKT with the survival and defense (anti-apoptotic effect) in trypanosomatid parasites [10,24,25]. Commonly, the AKT proteins or proteins within the PI3K/AKT/mTOR signaling pathway are the target of study for the development of pharmacological therapies against several types of cancer, since it has been demonstrated that these proteins participate in biological processes which are key for the cell, such as growth, differentiation, and survival [26]. The PH domain of the AKT proteins plays a crucial role, since it is responsible for the identification and transport of lipid substrates as phosphatidylinositol bisphosphate (PI2P) and phosphatidylinositol triphosphate (PI3P) produced by PI3K protein. These substrates are necessary for the correct intracellular signaling downstream. Therefore, AKT becomes a mediator of the activity of PI3K and is key for the control of this type of intracellular pathways [27,28].

Three PKB or AKT proteins have been found in humans with high sequence identity among them, and linked to molecular functions involved in metabolism, apoptosis, and proliferation. It has also been described that there are diverse cellular locations of these proteins in mice and humans, suggesting different routes of action [29,30]. To date, there is no information of AKT orthologs in *T. cruzi*. However, based on the above information, and the fact that we found one AKT-*like* protein different (UniProtKB ID Q4DL90) to the AKT-*like* protein suggested by us (UniProtKB ID Q4D6D3) [11], we propose that in *T. cruzi*, isoforms of this type of proteins could exist that can be associated with different cellular processes within the parasite. In addition, both proteins belong to different sections of the parasite genome (contigs) that has not been completely assembled, but with enough differences to consider them two individual gene products [31]. For example, the two sequences have an overall 45% of identity, but both shared the same functional domains (PH domain, kinase domain, and AGC kinase domain). However, part of the divergence is related to a *zinc fingers* domain found in one of the AKT-*like* proteins (UniProtKB ID Q4DL90) (Figure S1). This domain is categorized as a transporter of PI3P and key factor for the synthesis of ribosomal RNA, thus becoming regulatory factors of transcription processes in eukaryotic cells [32,33]. Something interesting is that *zinc fingers* domains are not present in any of the three human AKT proteins, and the sequence found in *Tc*AKT-*like* has a maximum identity of 41% with *zinc fingers* domains of humans. The above clearly opens the possibility to venture into the rational design of compounds that take advantage of these differences between the parasite and the human.

**Figure 5:**
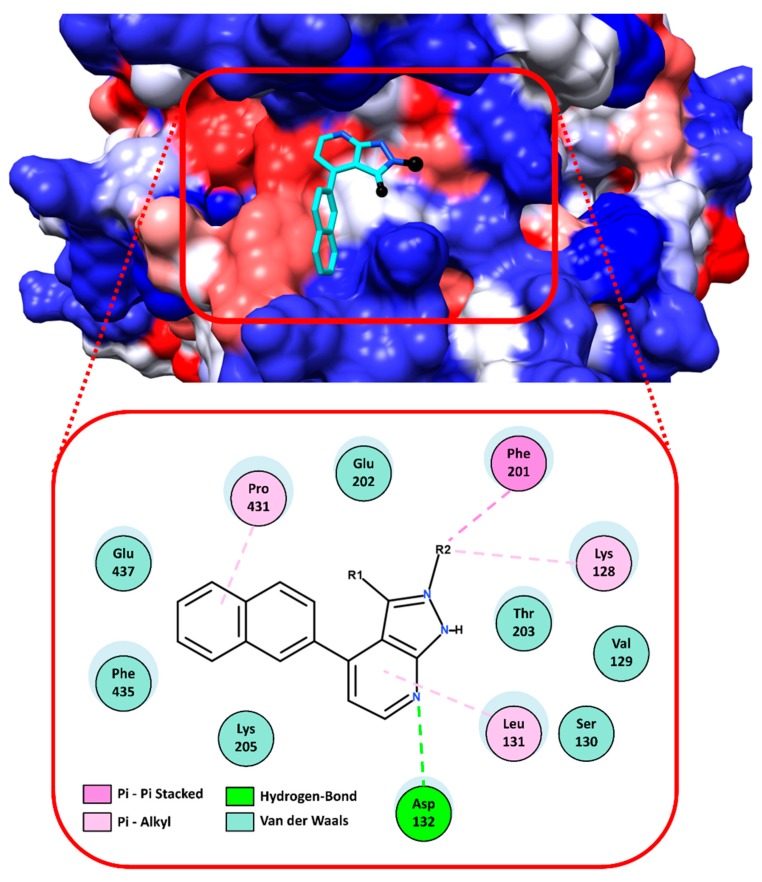
Interactions between the **UBMC-6** compound and the *Tc*AKT-*like* protein. In the upper part, the docking of the compound in the predicted binding site is observed in 3D. The compound **UBMC-6** is shown in cyan color and its fragments called R1 and R2 are shown in black; the protein is shown on a hydrophobic surface, where red is hydrophobic and blue is hydrophilic. In the lower part, the 2D interactions between the **UBMC-6** compound and the *Tc*AKT-*like* protein are shown. The blue shadows on the amino acids represent that they are or not on the surface of the protein; the larger the size, the more exposed it is.

Regarding the virtual screening approach, it allowed us to select a group of eight compounds with potential affinity for a pocket close to the PH domain of the *Tc*AKT-*like* protein (UniProtKB ID Q4D6D3). According to the in vitro results reported in Table 2, the interactions and the structural activity relationship of the **UBMC-6** compound were analyzed, since it was the compound that presented the best activity against amastigotes of *T. cruzi*. The 2D interactions of the compound **UBMC-6** are shown in Figure 5. The fragments **R1** and **R2** correspond to hydrophilic and aromatic rings substituted with heteroatoms groups, respectively. These fragments would facilitate the generation of hydrophobic interactions, Pi interactions, and possible hydrogen bonds. It is important to clarify that the **UBMC-6** scaffold is shared with other compounds from the list, but their chemical structures are not depicted because they are currently subjected to additional SAR studies and patent evaluations.

The compound **UBMC-6** has in its structure a pyrazolopyridine core that have been previously assayed against *T. cruzi* amastigotes, with an IC_50_ of 10.47 μM [34]. In addition, its structural analogue pyrazolopyrimidine has presented promising effects against apicomplexan parasites, such as *Neospora caninum* and *Toxoplasma gondii,* acting on the protein kinase CDPK in both cases [35,36]. Some analog compounds of the pyrazolopyridine nucleus have already been used in therapies against cancers, targeting the PH domain of the AKT proteins [37]. These data suggest that the compound **UBMC-6** could be a clear candidate for the study of new derivatives based on its structure. As a complementary objective of this investigation, the biological activity against *Leishmania braziliensis* of the selected compounds was also evaluated. Interestingly, the compound **UBMC-6** was also the one with the best results (i.e., IC_50_ of 18.71 μM). The results of these in vitro tests are summarized in Table S2. We are aware that the experimental validations were measured on cells and not directly on the recombinant protein. However, this is a first validation result against the parasite that will be crucial for further experiments able to demonstrate the direct interaction of the compounds with the AKT-*like* protein of *T. cruzi* and *L. braziliensis*.

Another conclusion of the docked compounds is that while they do not interact directly with any residue of the PH domain, they do interact with residues from a pocket close to it. These interactions could cause possible allosteric inhibition of the *Tc*AKT-*like* protein. The discovery of possible allosteric sites opens the opportunity to design new inhibitors against *Tc*AKT-*like*, increasing the specificity and reducing the classic risks of drug promiscuity. It is evident that more robust molecular studies, both experimental and computational, are necessary to clarify the possible effect of **UBMC-6** on the *Tc*AKT-*like* protein.

Finally, regarding other potential molecular targets from the protein–protein interaction network of *T. cruzi,* we can highlight the topoisomerase 2 protein, supported also by previous studies of this protein as a molecular target for antibacterial and anticancer treatment [38,39]. They have also been inhibited by compounds derived from the quinolone core, such as ciprofloxacin, levofloxacin, norfloxacin, and moxifloxacin, among others [40,41,42]. Interestingly, these compounds with antibacterial activity have shown to be promising to explore possible antiparasitic treatments [43,44].

## 4. Materials and Methods

### 4.1. Structural Modeling of the AKT-Like Protein of T. cruzi

The structural model of the AKT protein was obtained by a threading modeling approach using the I-TASSER web server (https://zhanglab.ccmb.med.umich.edu/I-TASSER/) [45]. The amino acid sequence of the *T. cruzi* AKT-*like* protein (*Tc*AKT-*like*) was obtained from UniProtKB with id Q4D6D3 [11]. The quality of the structural model was inspected using PDBsum (http://www.ebi.ac.uk/thornton-srv/databases/pdbsum/Generate.html) [46] and ProSA–web (https://prosa.services.came.sbg.ac.at/prosa.php) [47]. The metaPocket 2.0 (http://projects.biotec.tu-dresden.de/metapocket/) [19] and PockDrug (http://pockdrug.rpbs.univ-paris-diderot.fr) [48] web servers were used to predict possible cavities in the protein structure, prioritizing those located near or on their Pleckstrin Homology domain (PH). The predicted cavities were used as reference for the virtual screening.

### 4.2. Virtual Screening and Molecular Docking Simulations

The virtual screening was performed using the DrugDiscovery@TACC web portal (https://drugdiscovery.tacc.utexas.edu) [17] of the Texas Advanced Computing Center (TACC). The selected compound library contains ~640,000 drug-like molecules previously filtered. The receptor and the ligand structures were prepared following the protocol of software Autodock Tools v1.5.6 [49], which consists of adding polar hydrogens, calculating partial charges, and for the ligands, assigning rotatable bonds to confer flexibility. The screening was configured to search ligand poses in a box of 26 Å^3^ that covers the PH domain.

The first 8 compounds were selected for performing an additional molecular docking with the Swiss-Dock web server (http://www.swissdock.ch/) [50]. The two scoring functions from AutoDock Vina [51] and SwissDock were used in order to improve the predicted affinities and correlate them with the in vitro tests results.

### 4.3. In Silico Prediction of Toxicological Risks

Predictions of some toxicological risks for the selected compounds were made using the software DataWarrior v4.7.2 [52] and the web servers ProToxII (http://tox.charite.de/protox_II/) [53] and CarcinoPred-EL (http://ccsipb.lnu.edu.cn/toxicity/CarcinoPred-EL) [54]. The predicted toxic risks were Irritant, Mutagenic, Reproductive, Tumorigenic, Carcinogenic, Cytotoxic, and Immunotoxic effects. Compounds without any risk after the prediction of toxicity were classified as Negative, and those with at least one risk of toxicity were classified as Positive. Those compounds with a Negative classification have a higher probability to be effective in subsequent in vitro and in vivo experiments.

### 4.4. In Vitro Assay of the Antitrypanosomal Activity of the UBMC Compounds

The compounds were purchased from Molport (https://www.molport.com). The molecules were renamed using the UBMC tag in increasing order and solubilized in DMSO at a stock concentration of 20 mM.

A human promonocitic U937 line was cultivated in RPMI-1640 medium with 10% fetal bovine serum (FBS) at a concentration of 0.25 × 10^6^ cells/mL; the cells were transformed into macrophages by being incubated with phorbol 12-myristate 13-acetate (PMA) at 0.1 µg/mL. To adhere the cells, 0.1 mL of cells was added in 96-well dishes. Adherence was carried out for 72 h, and then epimastigote were added in the stationary phase of the Tulahuen strain producing β-galactosidase, at 5 parasite/cellU937 concentrations for 24 h at 37 °C with 5% CO_2_. To calculate the IC_50_ (Table 2), different concentrations of the compounds to evaluate and Benznidazole were added at an initial concentration of 20 µg/mL and 6 serial dilutions were made as a control of the effectiveness of the experiment, in addition to the control of the viability of the parasites (cells without drug treatment), from the blank of the culture medium (medium without cells), in duplicate. The cells were incubated for 72 h at 37 °C with 5% of CO_2_. All medium was removed and 100 µL of substrate for β-galactosidase diluted in PBS (chlorophenol β-d-galactopyranoside-CPRG network at 100 mM and 0.1% Nonidet P-40) was added to each well and incubated at 37 °C for 3 h. The colorimetric reaction was read at 570 nm and optical densities (OD) for each experimental condition were registered. The in vitro antitrypanosomal activity was defined by the reduction of the amount of amastigotes in infected cells (percentage of infection) following formula: (%) inhibition of infection = 1 − [(OD treated cells/OD untreated cells) × 100], where the OD of untreated cells corresponds to 100% of the infection. The OD of the blank was subtracted from the culture medium. The half inhibitory concentration (IC_50_) was calculated by the Probit method using the % of inhibition for each concentration [55].

The antitrypanosomal activity of each compound was rated according to its IC_50_. Thus, IC_50_ values <25 µM showed high activity, whereas IC_50_ > 25 µM and < 50 µM had moderated activity, and IC_50_ > 50 µM had low activity.

### 4.5. In Vitro Assay of Cytotoxicity of UBMC Compounds

The cytotoxicity of the compounds was evaluated according to the ability to kill macrophages derived from human monocytes (*h*MDM) by the macrophage-to-myofibroblast transition (MMT) method, following the procedures performed by Pastrana Restrepo et al. [56]. The *h*MDM were obtained from 50 mL of desfribrinated whole blood from healthy donors. These samples were mixed in a 1:1 ratio with Dulbecco PBS free of calcium and magnesium (DPBS). The mixture was centrifuged in a Ficoll Hypaque 1077 density gradient in a 1:3 ratio (blood–Ficoll) for separation of mononuclear cells, centrifuging at 2000 rpm for 20 min at 37 °C. The mononuclear cell layer was separated and these cells were washed twice with a solution of DPBS centrifuged at 13000 rpm for 10 min. After the last wash, the cells were resuspended in an RPMI medium with 10% autologous serum at a concentration of 0.3 × 10^6^ cells/mL. Then, 1 mL of cells was placed in each well of 24-well culture dishes and incubated at 37 °C, 5% CO_2_ for 72 h to allow the differentiation of monocytes to macrophages. For the MTT test, the *h*MDM were adjusted to a concentration of 0.5 × 10^6^ cells/mL in the RPMI medium supplemented with 10% FBS, and the concentration of the compound in the culture was adjusted (four serial dilutions starting at 200 μM). Amphotericin B and Doxorubicin were used as controls for the determination of cytotoxicity. Each experiment was performed in triplicate.

In this case, the in vitro cytotoxicity was defined by the decrease in the cell viability and growth, obtained from the OD for each experimental condition, using the following formula: (%) viability inhibition = 1 − [(OD treated cells/OD untreated cells) × 100], where the OD of untreated cells corresponds to 100% of the viability. Growth inhibition percentage data obtained for each experimental condition were used to calculate the half lethal concentration (LC_50_) by the Probit analysis [55].

The cytotoxicity of each compound was rated according to its LC_50_. Thus, LC_50_ values <100 μM were considered highly cytotoxic, whereas LC_50_ > 100 μM and < 200 μM were considered moderately cytotoxic and LC_50_ > 200 μM were considered as potentially noncytotoxic.

### 4.6. Reconstruction of the Protein–Protein Interaction Network of T. cruzi

A set of proteins corresponding to the CL Brener strain of *T. cruzi* was obtained from the UniProtKB database [57]. In parallel, information about protein–protein interactions in *T. cruzi* and species related to the genus trypanosomatidae (*Trypanosoma* and *Leishmania*) was downloaded from multiple repositories associated to the Intact database [58]. Interactions directly implicated with *T. cruzi* or previously predicted as interologs of other species were added directly to the network. For the other species, an ortholog inference analysis was performed to predict the presence of these interactions in *T. cruzi*. The Cytoscape v3.6.1 software was used for the visualization of the interaction networks [59]. In addition, the degree of connectivity by number of nodes was analyzed to study the scale-free topology of the built network.

### 4.7. Detection of Orthologs between T. cruzi and Those Reported in the PI3/AKT/mTOR Pathway

Given the important role of the AKT protein in signaling pathways in the host and in the enzymatic machinery provided by the parasite, a mapping of the proteins reported in the PI3K/AKT/mTOR pathway with the set of *T. cruzi* CL Breiner strain proteins was carried out. The pathway components were obtained from the KEGG database [60]. The comparison between sequences was done with a *Best Reciprocal Hits* protocol using the BLAST algorithm. Additionally, we calculated different topological metrics, including the degree of connectivity for each node, using the Network Analyzer plugin of Cytoscape v3.6.1 [59]. We also identified and suggested other proteins within the PI3K/AKT/mTOR signaling pathway that interact closely with the AKT protein of *T. cruzi,* and consequently, can be considered as molecular targets for further drug discovery studies against *T. cruzi*.

## 5. Conclusions

The computational structure-based strategy followed in this work was essential to identify a possible pocket near the PH domain of the *Tc*AKT-*like* protein, and subsequently identify compounds able to interact on the site, which were evaluated through in vitro tests. These findings could facilitate the design of effective compounds against trypanosomatids such as *T. cruzi* in future research. Although most of the compounds presented considerable cytotoxicity on host cells, compounds with better activities and low cytotoxicity can be taken as a starting point for the rational design of therapeutic agents against Chagas disease. In addition, based on the analysis of protein–protein interaction, we can conclude that the *Tc*AKT-*like* protein of *T. cruzi* is a promising candidate to be a molecular target for this and similar parasites.

## Figures and Tables

**Table 1:** Predicted scores and possible toxicological risks of compounds selected against *Trypanosoma cruzi*.

Compound	Predicted Scores (kcal/mol)		Predicted Toxicity
	AutoDock Vina	SwissDock	
UBMC-1	−10.6	-8.61	Positive
UBMC-2	−10.3	−8.33	Positive
UBMC-3	−10.0	−7.41	Positive
UBMC-4	−9.9	−8.41	Positive
UBMC-5	−9.8	−7.9	Negative
UBMC-6	−9.8	−7.63	Negative
UBMC-7	−9.7	−7.07	Positive
UBMC-8	−9.3	−7.27	Negative
Benznidazole	−6.2	−6.48	Positive

**Table 2:** Results of the in vitro assays of the chosen compounds with possible anti-*T. cruzi* activity.

Compound	IC_50_ (µM) ^a^	LC_50_ (µM) ^b^	SI ^c^
UBMC-6	14.25 ± 1	34.11 ± 3.14	2.39
UBMC-8	18.26 ± 1.30	55.2 ± 2.25	3.02
UBMC-7	19.44 ± 0.35	62.3 ± 5.63	3.2
UBMC-1	34.92 ± 3.87	61.66 ± 456	1.76
UBMC-3	37.53 ± 2.65	197.9 ± 7.84	5.27
UBMC-2	62.44 ± 4.62	44.5 ± 2.88	0.71
UBMC-5	69.82 ± 9.23	43.25 ± 7.53	0.61
UBMC-4	72.13 ± 8.58	70.88 ± 6.33	0.98
Benznidazole	16.61 ± 4	>200 (µg/mL)	12.04
Doxorrubicin	NA ^d^	3.91 ± 0.40	NC ^e^

^a^ IC_50_ inhibitory concentration of *T. cruzi* amastigote; ^b^ LC_50_ lethal concentration on human monocyte-derived macrophages (*h*MDMD); ^c^ SI selectivity index between *h*MDMD and amastigote; ^d^ NA not applicable; ^e^ NC not calculated. Data represent the mean value ± standard deviation

## Supplementary Materials

Supplementary materials can be found at http://www.mdpi.com/1422-0067/19/12/3951/s1.

## Abbreviations

PKB	Protein kinase B
RAC	Related to A- and C-kinases
PH	Pleckstrin homology domain
PI2P	Phosphatidylinositol bisphosphate
PI3P	Phosphatidylinositol trisphosphate
*Tc*AKT-*like*	AKT-*like* protein of *Trypanosoma cruzi*
OD	Optical density
