# Supplementary material for: Search of Allosteric Inhibitors and Associated Proteins of an AKT-like Kinase from Trypanosoma cruzi
Source: Int J Mol Sci. 2018 Dec 8;19(12):3951. doi: 10.3390/ijms19123951 (PMC6321509; doi:10.3390/ijms19123951)
Supplement: Supplementary file 1 [file ijms-19-03951-s001.zip › supp-files_tcakt.docx]

Supplementary files


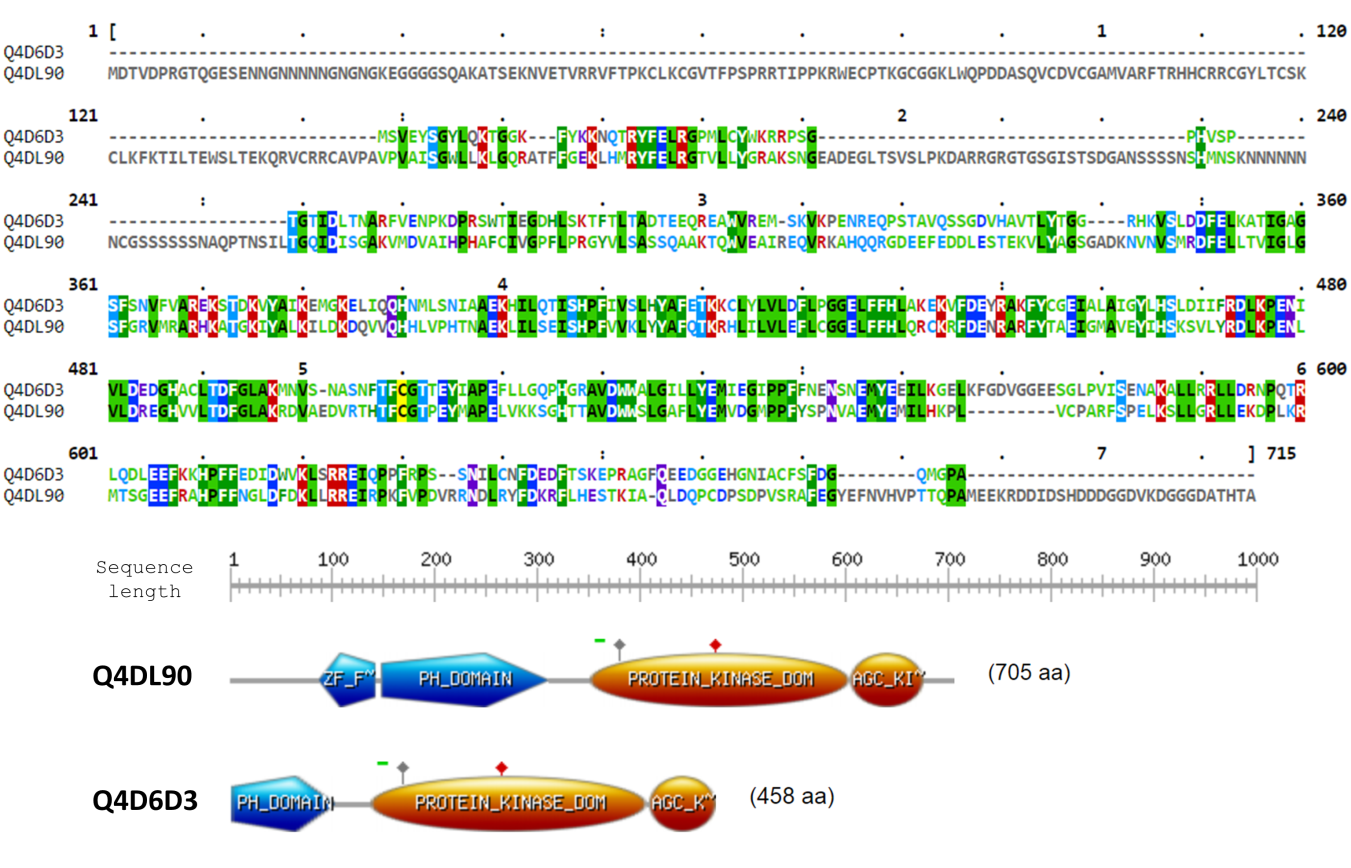


**Figure S1.** Sequence alignment between proteins with UniProtKB IDs Q4D6L90 and Q4D6D3. In the upper part, the sequential alignment between both proteins is observed. The identical amino acids between both sequences are highlighted with the same color. In the lower part, the functional domains present in both sequences are observed, corresponding to the possible isoforms of AKT-*like* in *T. cruzi*.

**Table S1.** Results of the toxicity risk predictions of the drug-like compounds selected.

| **Compound** | **Muta  (DW)** | **Tumo (DW)** | **Repr (DW)** | **Irrita (DW)** | **Canc (CP)** | **Imm (PTII)** | **Cytot (PTII)** |
| --- | --- | --- | --- | --- | --- | --- | --- |
| UBMC-12 | None | High | High | None | No | None | None |
| UBMC-10 | None | None | High | None | No | None | None |
| UBMC-9 | None | None | Low | None | No | None | None |
| UBMC-8 | High | None | None | None | No | None | None |
| UBMC-11 | None | None | None | None | No | None | None |
| UBMC-7 | None | None | None | None | No | None | None |
| UBMC-5 | None | None | None | High | No | None | None |
| UBMC-6 | None | None | None | None | No | None | None |
| Benznidazole | None | None | High | None | Yes | None | None |

DW: DataWarrior; CP: CarcinoPred-EL; PTII: ProToxII

**Table S2.** Results of the in vitro experiments of the selected compounds on *Leishmania braziliensis*.

| **Compound** | **IC_50_ (µM)^a^** | **LC_50_ (µM)^b^** | **SI^c^** |
| --- | --- | --- | --- |
|  |  |  |  |
| UBMC-1 | 40.68 ± 11.77 | 61.66 ± 456 | 1.51 |
| UBMC-2 | 63.6 ± 6.24 | 44.5 ± 2.88 | 0.70 |
| UBMC-3 | 468.53 ± 556.98 | 197.9 ± 7.84 | 0.43 |
| UBMC-4 | 131 ± 153.7 | 70.88 ± 6.33 | 0.54 |
| UBMC-5 | 32.34 ± 7.70 | 43.25 ± 7.53 | 1.34 |
| **UBMC-6** | **18.71 ± 1.82** | **34.11 ± 3.14** | **1.82** |
| UBMC-7 | 25.61 ± 2.24 | 62.3 ± 5.63 | 2.43 |
| UBMC-8 | 44.84 ± 7.15 | 55.2 ± 2.25 | 1.23 |
| Amphotericin B | 0.3 ± 0.07 | 49.78 ± 6.82 | - |

^a^ IC_50_ inhibitory concentration of *T. cruzi* amastigote.

^b^ LC50 lethal concentration on human monocyte-derived macrophages (*h*MDMD)

^c^ SI selectivity index between *h*MDMD and amastigote

Data represent the mean value ± standard deviation

**Table S3.** List of human proteins associated with the PI3K/AKT/mTOR pathway mapped against annotated proteins of *T. cruzi* strain CL Breiner.

| **Human UniProtKB ID** | **Human UniProtKB Gene** | **Gene Kegg** | ***T. cruzi* UniProtKB ID** | ***T. cruzi* GeneDB ID** |
| --- | --- | --- | --- | --- |
| Q12923 | PTN13_HUMAN | PTPN13 | Q4D9I2 | 353153.XP_811026.1 |
| **Q9Y243** | **AKT3_HUMAN** | **AKT3** | **Q4D4Z9** | **353153.XP_809454.1** |
| **P31751** | **AKT2_HUMAN** | **AKT2** | **Q4DL90** | **353153.XP_815140.1** |
| P07384 | CAN1_HUMAN | CAPN1 | Q4DQN6 | 353153.XP_816696.1 |
| Q71U36 | TBA1A_HUMAN | TUBA1A | Q4CLA1 | 353153.XP_802499.1 |
| Q14643 | ITPR1_HUMAN | ITPR1 | Q4D7M2 | 353153.XP_810378.1 |
| P42338 | PK3CB_HUMAN | PIK3CB | Q4CUM7 | 353153.XP_805828.1 |
| P05198 | IF2A_HUMAN | EIF2S1 | Q4E620 | 353153.XP_822107.1 |
| P36507 | MP2K2_HUMAN | MAP2K2 | Q4E0A0 | 353153.XP_820028.1 |
| O14727 | APAF_HUMAN | APAF1 | Q4DPC6 | 353153.XP_816222.1 |
| P60709 | ACTB_HUMAN | ACTB | Q4CYQ8 | 353153.XP_807262.1 |
| P99999 | CYC_HUMAN | CYCS | Q4CV48 | 353153.XP_806001.1 |
| Q9NZJ5 | E2AK3_HUMAN | EIF2AK3 | Q4CTA4 | 353153.XP_805355.1 |
| Q14249 | NUCG_HUMAN | ENDOG | Q4DF49 | 353153.XP_813011.1 |
| P01116 | RASK_HUMAN | KRAS | Q4DUQ2 | 353153.XP_818107.1 |
| P07858 | CATB_HUMAN | CTSB | Q4DQB0 | 353153.XP_816569.1 |
| P27361 | MK03_HUMAN | MAPK3 | Q4CZQ7 | 353153.XP_807605.1 |
| P25963 | IKBA_HUMAN | NFKBIA | Q4D7G1 | 353153.XP_810311.1 |
| Q99683 | M3K5_HUMAN | MAP3K5 | Q4D1T8 | 353153.XP_808337.1 |
| Q9UGN5 | PARP2_HUMAN | PARP2 | Q4D1I2 | 353153.XP_808238.1 |

**Table S4.** Data of the topological metrics of connectivity (degree) and betweenness centrality for the mapped proteins of *T. cruzi* strain CL Breiner in the PI3K/AKT/mTOR pathway.

| ***T. cruzi* UniProtKB ID** | ***T. cruzi* GeneDB ID** | **Degree** | **Betweeness** |
| --- | --- | --- | --- |
| Q4D9I2 | 353153.XP_811026.1 | 41 | 3.33E-06 |
| Q4D4Z9 | 353153.XP_809454.1 | 81 | 8.77E-04 |
| **Q4DL90** | **353153.XP_815140.1** | **89** | **0.002039** |
| Q4DQN6 | 353153.XP_816696.1 | NO | NO |
| Q4CLA1 | 353153.XP_802499.1 | 135 | 0.007609 |
| Q4D7M2 | 353153.XP_810378.1 | 9 | 4.23E-04 |
| Q4CUM7 | 353153.XP_805828.1 | 54 | 4.90E-04 |
| Q4E620 | 353153.XP_822107.1 | 188 | 0.001311 |
| Q4E0A0 | 353153.XP_820028.1 | 99 | 2.53E-04 |
| Q4DPC6 | 353153.XP_816222.1 | 19 | 1.48E-05 |
| Q4CYQ8 | 353153.XP_807262.1 | 124 | 0.002288 |
| Q4CV48 | 353153.XP_806001.1 | 16 | 6.07E-04 |
| Q4CTA4 | 353153.XP_805355.1 | 10 | 2.92E-06 |
| Q4DF49 | 353153.XP_813011.1 | 8 | 2.81E-04 |
| Q4DUQ2 | 353153.XP_818107.1 | 126 | 8.96E-04 |
| Q4DQB0 | 353153.XP_816569.1 | NO | NO |
| Q4CZQ7 | 353153.XP_807605.1 | 166 | 0.001923 |
| Q4D7G1 | 353153.XP_810311.1 | NO | NO |
| Q4D1T8 | 353153.XP_808337.1 | 53 | 1.07E-05 |
| Q4D1I2 | 353153.XP_808238.1 | 31 | 2.83E-04 |

**Table S5.** List of proteins of the protein­­­–protein interaction network of *T. cruzi* CL Breiner strain ranged based on the degree within the network.

| **ID** | **Average Shortest Path** | **Degree** | **UniProtKB ID** | **Function** |
| --- | --- | --- | --- | --- |
| 353153.XP_810722.1 | 2.31819108 | 740 | Q4D8L4_TRYCC | DNA topoisomerase 2 |
| 353153.XP_812644.1 | 2.31819108 | 740 | Q4DE53_TRYCC | DNA topoisomerase 2 |
| 353153.XP_813815.1 | 2.31819108 | 740 | Q4DHG5_TRYCC | DNA topoisomerase 2 |
| 353153.XP_820295.1 | 2.31819108 | 740 | Q4E0W1_TRYCC | DNA topoisomerase 2 |
| 353153.XP_820425.1 | 2.47565696 | 462 | Q4E1D4_TRYCC | GMP synthase, putative |
| 353153.XP_817140.1 | 2.89814626 | 417 | Q4DRX6_TRYCC | 60S acidic ribosomal protein, putative |
| 353153.XP_815327.1 | 2.51843553 | 392 | Q4CKH4_TRYCC | Dihydrofolate reductase-thymidylate synthase, putative (Fragment) |
| 353153.XP_805668.1 | 2.73518028 | 390 | Q4CU60_TRYCC | 60S ribosomal protein L5, putative |
| 353153.XP_814693.1 | 2.73518028 | 390 | Q4CU61_TRYCC | 60S ribosomal protein L5, putative |
| 353153.XP_805815.1 | 2.75718069 | 384 | Q4CUL0_TRYCC | 40S ribosomal protein S3, putative |
| 353153.XP_816670.1 | 2.75718069 | 384 | Q4DMK9_TRYCC | 40S ribosomal protein S3, putative |
| 353153.XP_805319.1 | 2.88103483 | 381 | Q4CT69_TRYCC | Uncharacterized protein |
| 353153.XP_811660.1 | 2.88103483 | 381 | Q4DBC1_TRYCC | Uncharacterized protein |
| 353153.XP_814674.1 | 2.77490324 | 379 | Q4D6P8_TRYCC | Ribosomal protein L13, putative (Fragment) |
| 353153.XP_806665.1 | 3.08698309 | 377 | Q4CX08_TRYCC | 40S ribosomal protein S8, putative |
| 353153.XP_818615.1 | 3.08698309 | 377 | Q4DW55_TRYCC | 40S ribosomal protein S8, putative |
| 353153.XP_820220.1 | 3.09859442 | 374 | Q4E0Q9_TRYCC | 60S ribosomal protein L24, putative |
| 353153.XP_821233.1 | 3.09859442 | 374 | Q4E3N7_TRYCC | 60S ribosomal protein L24, putative |
| 353153.XP_814355.1 | 2.79486657 | 359 | Q4DIZ9_TRYCC | 40S ribosomal protein S2, putative |
| 353153.XP_803249.1 | 2.74781014 | 352 | Q4CNF1_TRYCC | 60S ribosomal protein L11, putative (Fragment) |
| 353153.XP_805772.1 | 2.56325117 | 352 | Q4CQ93_TRYCC | Inosine-5'-monophosphate dehydrogenase, putative (Fragment) |
| 353153.XP_815489.1 | 2.56325117 | 352 | Q4DM82_TRYCC | Inosine-5'-monophosphate dehydrogenase |
| 353153.XP_820501.1 | 2.56325117 | 352 | Q4E1M2_TRYCC | GMP reductase |
| 353153.XP_814064.1 | 2.99348136 | 351 | Q4DI49_TRYCC | 60S ribosomal protein L23a, putative |
| 353153.XP_820993.1 | 2.99348136 | 351 | Q4E2Y1_TRYCC | 60S ribosomal protein L23a, putative |
| 353153.XP_810859.1 | 2.85149725 | 345 | Q4D904_TRYCC | ATP-dependent DEAD/H RNA helicase, putative |
| 353153.XP_809990.1 | 2.84925647 | 342 | Q4D6H7_TRYCC | Ribosomal protein S20, putative |
| 353153.XP_810552.1 | 2.8883683 | 340 | Q4CWD6_TRYCC | 40S ribosomal protein S13, putative |
| 353153.XP_811944.1 | 2.8883683 | 340 | Q4DC38_TRYCC | 40S ribosomal protein S13, putative |
