# Supplementary figures and images for: Search of Allosteric Inhibitors and Associated Proteins of an AKT-like Kinase from Trypanosoma cruzi
Source: Int J Mol Sci. 2018 Dec 8;19(12):3951. doi: 10.3390/ijms19123951 (PMC6321509; doi:10.3390/ijms19123951)

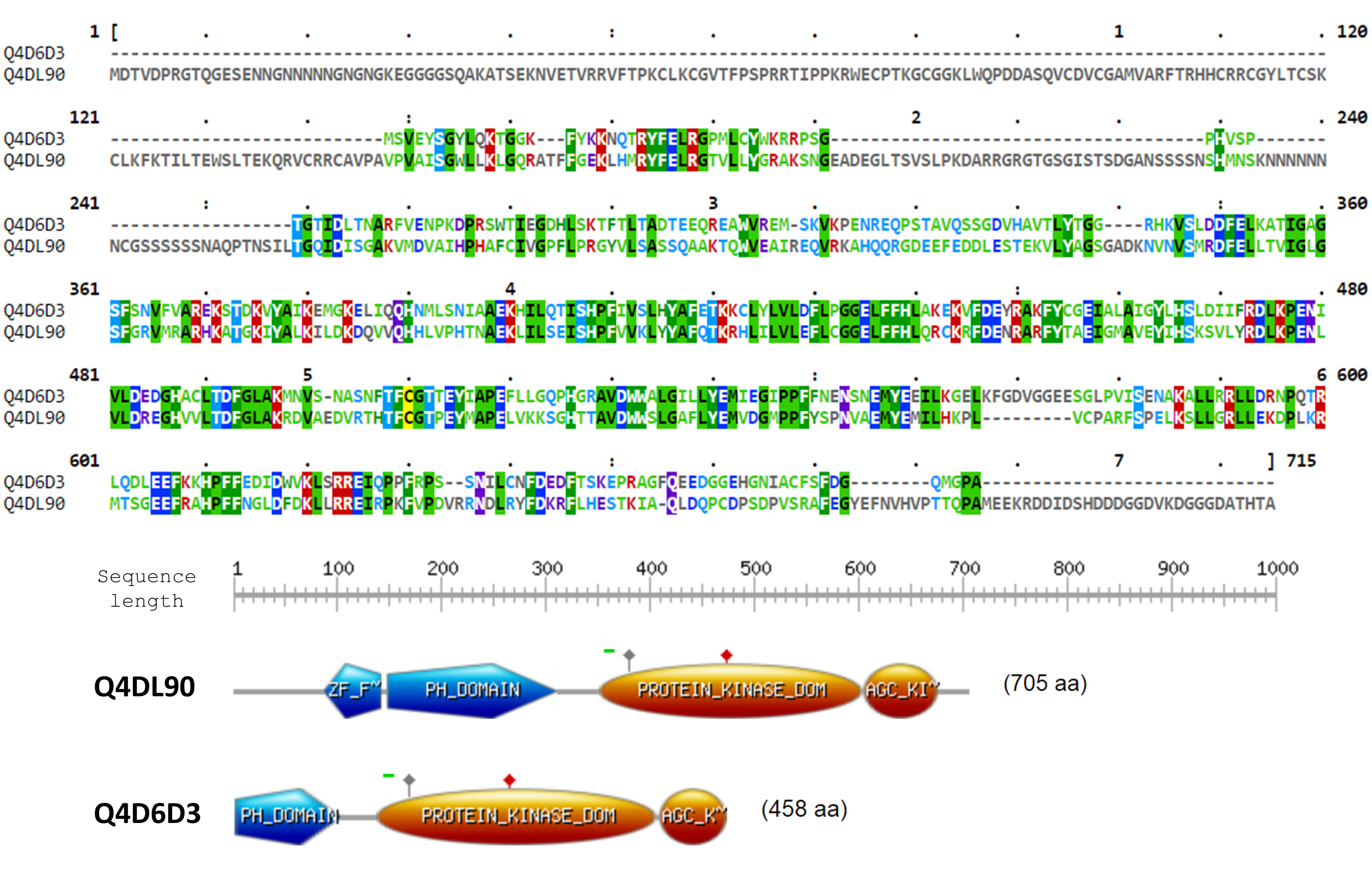

Supplement: Supplementary file 1 [file ijms-19-03951-s001.zip › figS1.tif]
